# Alpha-fetoprotein and APRI as predictive markers for patients with Type C hepatitis B-related acute-on-chronic liver failure: a retrospective study

**DOI:** 10.1186/s12876-024-03276-x

**Published:** 2024-06-04

**Authors:** Chunyan Li, Hao Hu, Chengzhi Bai, Huaqian Xu, Lin Liu, Shanhong Tang

**Affiliations:** 1 Department of Gastroenterology, The General Hospital of Western Theater Command, Chengdu, 610083 Sichuan China; 2 Endoscopy Center and Endoscopy Research Institute, Shanghai Collaborative Innovation Center of Endoscopy, Zhongshan Hospital, Fudan University, Shanghai, 200032 China

**Keywords:** Alpha-fetoprotein, Liver fibrosis, Type C HBV-ACLF, Prognostic score

## Abstract

**Background:**

Type C hepatitis B-related acute-on-chronic liver failure (HBV-ACLF), which is based on decompensated cirrhosis, has different laboratory tests, precipitating events, organ failure and clinical outcomes. The predictors of prognosis for type C HBV-ACLF patients are different from those for other subgroups. This study aimed to construct a novel, short-term prognostic score that applied serological indicators of hepatic regeneration and noninvasive assessment of liver fibrosis to predict outcomes in patients with type C HBV-ACLF.

**Method:**

Patients with type C HBV-ACLF were observed for 90 days. Demographic information, clinical examination, and laboratory test results of the enrolled patients were collected. Univariate and multivariate logistic regression were performed to identify independent prognostic factors and develop a novel prognostic scoring system. A receiver operating characteristic (ROC) curve was used to analyse the performance of the model.

**Results:**

A total of 224 patients with type C HBV-ACLF were finally included. The overall survival rate within 90 days was 47.77%. Age, total bilirubin (TBil), international normalized ratio (INR), alpha-fetoprotein (AFP), white blood cell (WBC), serum sodium (Na), and aspartate aminotransferase/platelet ratio index (APRI) were found to be independent prognostic factors. According to the results of the logistic regression analysis, a new prognostic model (named the A3Twin score) was established. The area under the curve (AUC) of the receiver operating characteristic curve (ROC) was 0.851 [95% CI (0.801-0.901)], the sensitivity was 78.8%, and the specificity was 71.8%, which were significantly higher than those of the MELD, IMELD, MELD-Na, TACIA and COSSH‐ACLF II scores (all P < 0.001). Patients with lower A3Twin scores (<-9.07) survived longer.

**Conclusions:**

A new prognostic scoring system for patients with type C HBV-ACLF based on seven routine indices was established in our study and can accurately predict short-term mortality and might be used to guide clinical management.

**Supplementary Information:**

The online version contains supplementary material available at 10.1186/s12876-024-03276-x.

## Background

Acute-on-chronic liver failure (ACLF) is a life-threatening clinical syndrome with rapid progression of hepatic injury based on chronic liver diseases, accompanied by liver and/or extrahepatic organ failure [1]. In the West, the most common aetiologies of ACLF are alcoholic liver disease and hepatitis C virus infection. In the East, hepatitis B virus (HBV) infections dominate [2], and HBV infection is the major cause of ACLF in China, accounting for more than 50% of cases [3]. ACLF can be induced by acute intrahepatic (e.g, alcoholic hepatitis or hepatitis B virus reactivation) or extrahepatic insults (e.g, bacterial infection, gastrointestinal haemorrhage). The ACLF insults also differ in Eastern and Western populations. Due to the differences in aetiology and inducement, ACLF has regional phenotypic specificities, and there are different definitions for ACLF in different geographic regions. One of the major differences is that the Asian Pacific Association for the Study of the Liver (APASL) includes noncirrhotic chronic liver disease (CLD) and compensated cirrhosis to represent “chronic”, whereas the European Association for the Study of the Liver (EASL) includes only cirrhosis, either compensated or decompensated, to define CLD [4]. Attempting to cover all ACLF patients diagnosed in the East and West, the WGO defined ACLF into three categories in 2014[5]: patients with CLD but no cirrhosis (type A), compensated cirrhosis (type B), and decompensated cirrhosis (type C).

Type-C ACLF patients with prior decompensated cirrhosis have the lowest baseline hepatic reserve, heaviest liver fibrosis, and highest portal hypertension (PH) [6]. Decompensated cirrhosis is associated with the development of disease-related complications, such as ascites, oesophageal variceal bleeding, and hepatic encephalopathy, usually with HVPG >10 mmHg. PH has strong positive implications for the patient’s disease course and prognosis [7]. Type C patients have higher mortalities than either type A or type B patients [8]. Except for liver transplantation, the current therapeutic methods are limited. Thus, prognostic models could play an essential role in type C ACLF management.

At present, there are many prognostic scoring models for ACLF, including the MELD score [9], CLIF-C ACLF score [10], AARC score [11], COSSH-ACLF score [12], and COSSH-ACLF II score [13]. The predictive accuracy of MELD is limited [14]. Based on the complicated assessment of organ failure, the CLIF-C ACLF and COSSH-ACLF scores should be further simplified. The AARC score and COSSH-ACLF II score contain subjective indicators. They rarely focus on liver fibrosis/portal hypertension and liver regeneration. This study aimed to construct a new, short-term prognosis model that considers the combination of noninvasive assessment of liver fibrosis and liver regeneration to provide a simple and accurate prognosis of type C HBV-ACLF.

## Methods

### Patient management

Standard medical treatment was obtained, including bed rest, liver-protective treatment, and energy supplements. Patients also received plasma and albumin infusion, water-electrolyte maintenance, and complication-preventing treatment. All patients received antiviral therapy.

### Study population

**Fig. 1:**
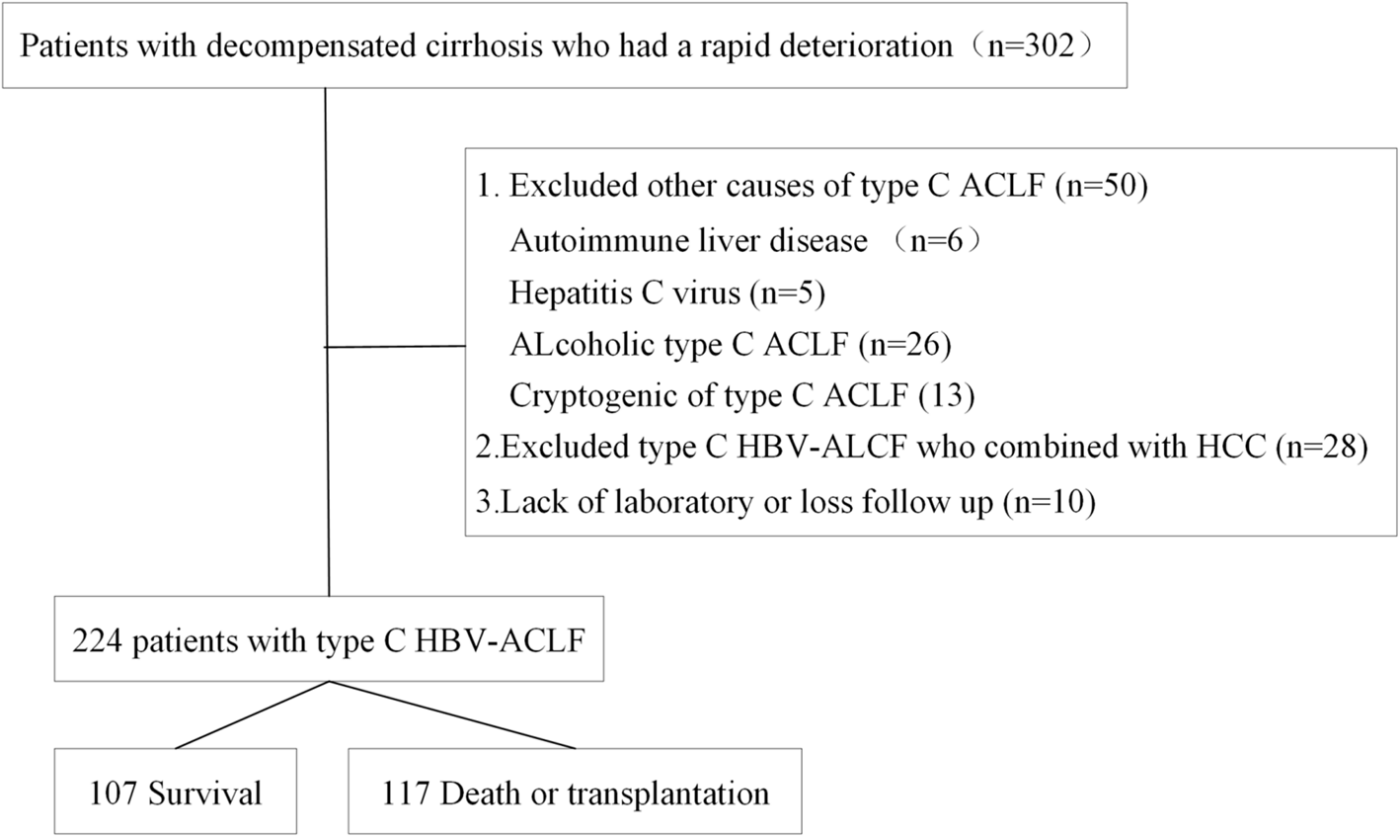
Inclusion and exclusion criteria of this research. HBV-ACLF: hepatitis B-related acute-on-chronic liver failure; HCC: hepatocellular carcinoma

Patients were retrospectively screened and enrolled from January 2015 to January 2023 in the General Hospital of Western Theater Command and followed up for 90 days from the date of ACLF diagnosis. The endpoint of follow-up was death or liver transplantation. In this retrospective analysis, a total of 302 type C ACLF patients were initially screened. The diagnostic criteria for HBV-ACLF should include the following: (1) The cause of ACLF was hepatitis B virus (HBV). The diagnosis of chronic hepatitis B was hepatitis B surface antigen positivity or hepatitis B virus deoxyribonucleic acid level (HBV-DNA) positivity for > 6 months. (2) The patients were those who were previously diagnosed with hepatitis B virus-associated decompensated cirrhosis. Liver cirrhosis was diagnosed based on previous liver biopsy findings, ultrasonography, computed tomography, or magnetic resonance imaging findings. Decompensated cirrhosis was diagnosed based on cirrhosis with portal hypertension complications (ascites, hepatic encephalopathy (HE), gastrointestinal haemorrhage, bacterial infection, and hepatorenal syndrome, or any combination of these and/or hypo-hepatic function). (3) ACLF was diagnosed according to the Asian Pacific Association for the Study of the Liver (APASL) [15]: ACLF is an acute hepatic insult manifesting as jaundice (serum bilirubin ≥ 5 mg/dL (85 umol/L) and coagulopathy (INR ≥ 1.5 or prothrombin activity < 40%) complicated within 4 weeks by clinical ascites and/or encephalopathy in a patient with previously diagnosed or undiagnosed chronic liver disease/cirrhosis, and is associated with a high 28-day mortality. The exclusion criteria were as follows: (1) age below 18 or over 80 years; (2) coinfection with other viruses (hepatitis A, hepatitis C, hepatitis D or E, or HIV); (3) other causes of ACLF, such as autoimmune liver disease, drug-induced hepatitis, alcoholic liver injury, *etc*; (4) hepatocellular carcinoma (HCC); and (5) clinical data missing or patients lost to follow-up. Finally, 224 patients were included in this study (Fig. 1).

### Data collection

Patient demographics (age, sex) and laboratory data at the time of admission, including routine blood tests, liver and kidney function, coagulation parameters, blood lipids, serum tumour markers, HBV-DNA and hepatitis B e antigen (HBeAg), were collected. Complications such as ascites, gastrointestinal haemorrhage, hepatic encephalopathy (HE), acute kidney injury (AKI), and bacterial infections were also recorded and analysed. The aspartate aminotransferase/platelet ratio index (APRI) and Fibrosis-4 (FIB4) were used to assess the degree of liver fibrosis or cirrhosis. APRI=[(AST/ULN)×100]/PLT; FIB-4=(age ×AST)/(PLT×*ALT^1/2^. Prognostic models including the MELD, MELD-Na, iMELD, COSSH-ACLF II scores, and TACIA scores were recorded as tools of condition assessment. The MELD score was calculated by the following formula: MELD = 3.78 × ln [TBil (mg/dL)] + 11.2 × ln (INR) + 9.57 × ln [serum creatinine (mg/dL)] + 6.4 [16]. MELD Na = MELD – Na- [0.025 × MELD × (140 - Na)] + 140 [17]. iMELD=MELD+ (0.3×age)-(0.7× Na) +100 [18]. COSSH-ACLF II =1.649 × ln (INR) + 0.457 × HE score (HE grade: 0/1, 1-2/2 and 3-4/3) + 0.425 × ln (neutrophil) (109/L) + 0.396 × ln (TBil) (µmol/L) + 0.576 × ln (serum urea) (mmol/L) + 0.033 × age [13]. TACIA= 0.003 × TBil (µmol/L) + 0.036 × age + 0.009 × Cre (μmol/L) + 0.525 × INR – 0.003 × AFP (ng/mL) [19].

### Statistical analyses

SPSS version 25.0 software (IBM Corp, Armonk, NY, USA) was used for statistical processing. Continuous data were expressed as the means ± SDs or medians with an appropriate interquartile range. Those variables were compared by using Student's *t* test or the nonparametric Mann‒Whitney *U* test. Percentages were used to present categorical data, which were compared by the chi-squared test or Fisher's exact test. Binary logistic regression with forward elimination was employed to demonstrate the independent predictors for the 90-day mortality rate of patients with type C HBV-ACLF and establish a new prognostic scoring system. The Spearman correlation test was conducted to examine the correlation between AFP and other biochemical parameters. The area under the receiver operating characteristic curve (ROC) was used for model discrimination and calibration. Survival analysis was performed using Kaplan–Meier analysis, and differentiation analysis was evaluated by the log-rank test. The Youden index was used to identify the optimal cut-off point. Statistical significance was considered when *P* ≤ 0.05.

## Result

### Characteristics and outcomes of type C HBV-ACLF patients

There were 224 patients included in our study. The baseline characteristics of type C HBV-ACLF patients are shown in Table 1. The AFP (64.22 (16.10-178.61) vs. 24.80 (10.50-76.45), *p* < 0.05) of the survival group was significantly higher than that of the death or transplanted group, whereas the APRI (8.00 (4.37-14.91) vs. 10.90 (4.72-25.08), *p* < 0.05) was significantly lower than that of the death or transplanted group. The age, INR, TBil, WBC, neutrophils, Na, Cre, and complications of the survival group were significantly lower than those of the death or transplanted group (*p* < 0.05). Furthermore, there were no significant differences between the groups in terms of Alb, PLT, AST, ALT and HBV-DNA. During a 90-day follow-up, one hundred and seventeen patients (52.23%) were deceased or received a liver transplant, and the liver transplant-free survival rate was 47.77% (107/224).

**Table 1:** Clinical characteristics of type C HBV-ACLF patients

**Parameter**	**Survival (*n*=107)**	**Death or transplantation (*n*=117)**	***P *Value**
Age(years)	50.78±10.06	55.16±10.61	0.002
AFP(ng/mL)	64.22(16.10,178.61)	24.80(10.50,76.45)	0.004
Alb(g/L)	30.72±4.61	30.12±4.41	0.305
Pre-Alb(mg/L)	45.79±2.14	38.81±2.61	0.038
TBil(umol/L)	263.57(208.90,401.25)	405.00(294.32,516.44)	<0.001
ALT(U/L)	270.70(100.40,654.35)	274.65(86.85,694.18)	0.867
AST(U/L)	241.80(113.45,537.25)	274.00(124.70,710.40)	0.237
INR	1.72(1.55,2.39)	2.13(1.77,2.63)	<0.001
WBC(×10^9^/L)	5.5(3.82,7. 73)	6.94(5.11,9.73)	<0.001
PLT(×10^9^/L)	79.00(47.50,111.00)	68(47.25,99.00)	0.155
Neutrophil Count(×10^9^/L)	3.79(2.48,5.59)	5.32(3.74,7.55)	<0.001
Cre (μmol/L)	74.00(63.00,89.00)	83.00(67.25,108.80)	0.003
Na (mmo/L)	135.11±0.46	133.03±051	0.003
HBV-DNA(log10IU/mL)	5.09(3,80,6.47)	5.17(4.06,6.58)	0.875
NLR	4.12(2.75,6.90)	7.25(4.42,10.56)	<0.001
MLR	0.48(0.34,0.69)	0.84(0.59,1.19)	<0.001
APRI	8.00(4.37,14.91)	10.90(4.72,25.08)	0.033
FIB-4	10.23(5.37,19.82)	15.04(8.62,28.28)	0.001
Ascites (n %)	99(92.7)	115(98.3)	0.041
HE (n %)	11(10.3)	63(53.8)	<0.001
MELD	21.84(19.21,24.31)	26.28(23.09,29.70)	<0.001
MELD-Na	23.26(20.12,27.96)	29.70(24.50,36.28)	<0.001
iMELD	42. 26(38.95,45.65)	49. 48(44.40,55.56)	<0.001
COSSH-ACLF II	6.84±0.69	8.03±1.09	<0.001
TACIA	3.86±1.45	5.14±1.19	<0.001

*AFP* Alpha-fetoprotein, *Alb* Albumin, *Pre-Alb* Pre-albumin, *TBil* Total bilirubin, *ALT* Alanine aminotransferase, *AST* Aspartate aminotransferase, *INR* International normalized ratio, *WBC* White blood cell, *PLT* Platelet, Cre creatinine, *Na* serum sodium, *NLR* Neutrophil to lymphocyte ratio, *MLR* Monocyte-to-lymphocyte ratio, *APRI* Aspartate aminotransferase -to-platelet ratio index, *FIB-4* Fibrosis-4, *MELD* Model for end-stage liver disease, *MELD-Na* MELD-sodium, *iMELD* integrated MELD, *COSSH-ACLF II* Chinese Group on the Study of Severe Hepatitis B-ACLF II score

### Independent prognostic factors and development of a new predictive model

First, the clinical variables were included in the univariate logistic analysis. The results of the analysis showed that age, TBil, INR, AFP, WBC, Cre, Na, NLR, and APRI showed significant associations with 90-day survival (*p* < 0.05). Then, the choice of variables for the multivariable analysis was based on the results of the univariable analysis, as shown in Table 2. Age[OR1.05, 95% CI(1.00-1.09), *p* = 0.019], TBil[OR 1.01, 95% CI(1.00-1.01), *p* ≤0.001] , INR[OR 3.76, 95% CI (1.23-10.89), *p* =0.015], AFP[OR 1.00, 95% CI (0.99-1.00), *p* =0.026], WBC[OR 1.16, 95% CI (1.01-1.32), *p*=0.03], Na[OR 0.89, 95% CI(0.82-0.97), *p* =0.009]and APRI[OR 1.02, 95% CI (1.00-1.04), *p* =0.047] were independently associated with the prognosis of HBV-ACLF over 90 days. Then, a new prognostic model (we named it the A3Twin score) according to the β determination formula in the regression equation was established as the following mathematical formula: A3Twin=0.047×age+0.005×TBil+ 1.325×INR+0.147×WBC+0.019×APRI-0.111×Na-0.003×AFP-1.878.

**Table 2:** Univariate and multivariate logistic regression analyses of 90-day mortality

**Parameter**	**Univariate analyses**			**Multivariate analyses**		
	**β**	**OR(95%CL)**	***P***	**β**	**OR(95%CL)**	***P***
Age(years)	0.043	1.04(1.01,1.07)	0.001	0.047	1.05(1.00,1.09)	0.019
Pre-Alb(mg/L)	-0.012	0.99(0.98,1.00)	0.047			
TBil(umol/L)	0.005	1.01(1.00,1.01)	0.001	0.005	1.01(1.00,1.01)	0.001
ALT(U/L)	0.001	1.00(1.00,1.00)	0.471			
AST(U/L)	0.001	1.00(1.00,1.00)	0.115			
INR	1.739	5.95(3.07,11.54)	0.001	1.325	3.76(1.23,10.89)	0.015
AFP(ng/mL)	-0.002	0.99(0.99,1.00)	0.014	-0.003	1. 00(0.99,1.00)	0.026
WBC(×10^9^/L)	0.143	1.16(1.06,1.26)	<0.001	0.147	1.16(1.01,1.32)	0.030
PLT(×10^9^/L)	-0.006	0.99(0.98,1.00)	0.071			
Cre (μmol/L)	0.005	1.01(1.00,1.01)	0.048	0.002	1.00(0.99,1.01)	0.645
Na (mmo/L)	-0.078	0.93(0.88,0.98)	0.004	-0.111	0.89(0.82,0.97)	0.009
HBVDNA(log10IU/Ml)	0.045	1.02(0.96,106)	0.466			
NLR	0.057	1.06(1.00,1.12)	0.037	0.001	1.00(0.97,1.03)	0.645
APRI	0.014	1.02(1.00,1.03)	0.041	0.019	1.02(1.00,1.04)	0.047
FIB-4	0.015	1.02(1.00,1.03)	0.052			

### Correlations of AFP with biochemical parameters in patients with type C HBV-ACLF

We evaluated the correlations between AFP and clinical parameters in patients with type C HBV-ACLF. The results showed that the AFP level was significantly positively correlated with PLT (r = 0.198, *P* = 0.003), Pre-Alb (r = 0.146, *P* = 0.035), and ALT (r = 0.280, *P* <0.001) and negatively correlated with MLR (r = -0.152, *P* = 0.023) and FIB-4 (r = -0.270, *P*<0.001). However, there were no significant correlations among APF and TBil, AST, and APRI. (Table 3)

**Table 3:** Correlations of AFP with biochemical parameters in patients with type C HBV-ACLF

**AFP vs.**	**Spearman’s**	***P* value**
PLT	0.198	0.003
MLR	-0.152	0.023
Pre-Alb	0.146	0.035
TBil	0.155	0.055
ALT	0.280	<0.001
AST	0.099	0.141
FIB-4	-0.270	<0.001
APRI	-0.022	0.750

### Performance of the new model

**Fig. 2:**
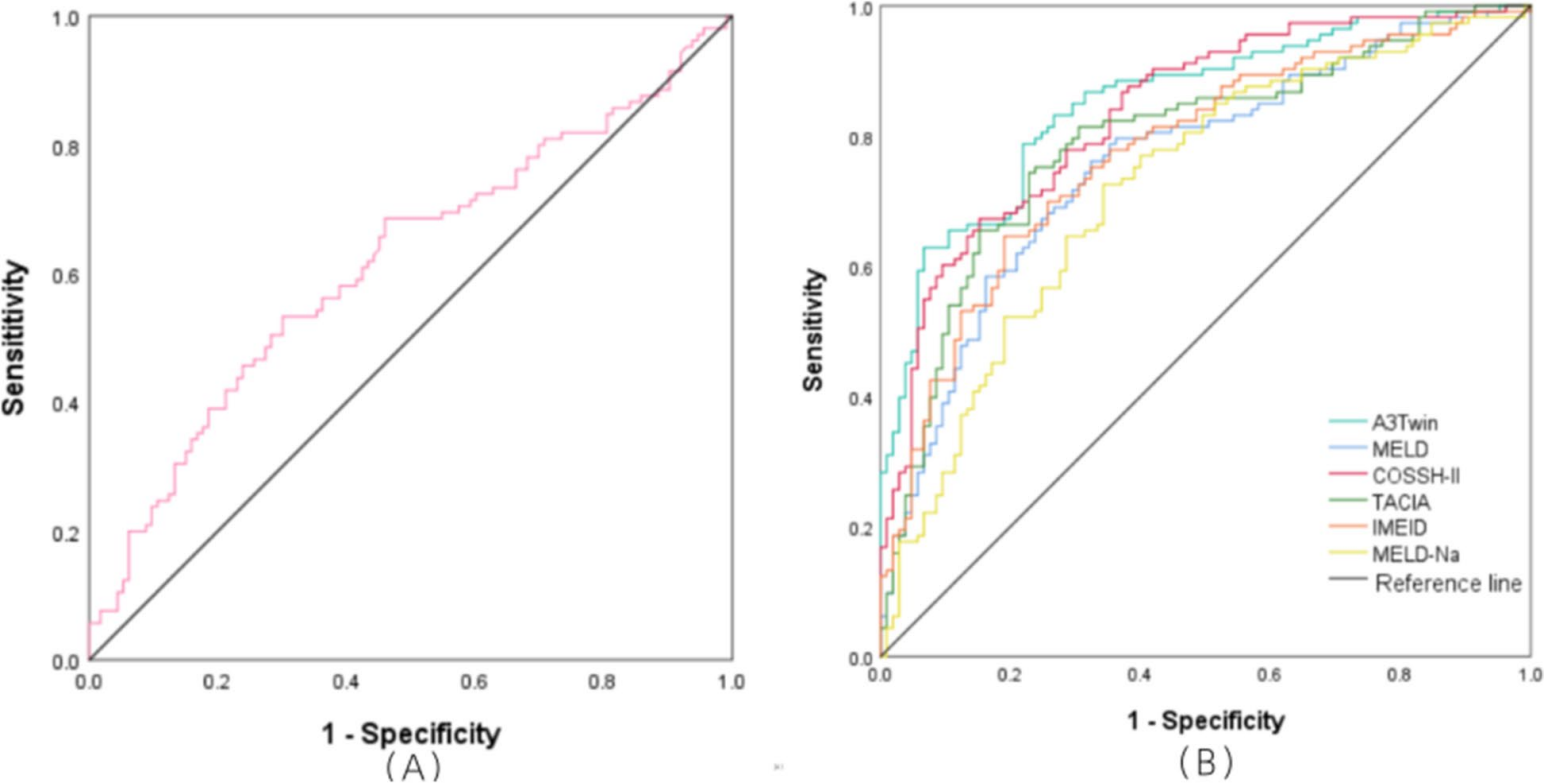
Efficacy of receiver operating characteristic curves (ROC) in predicting the outcome. (a)ROC curve of AFP. (b) ROC curve for the prognostic model. AFP: alpha-fetoprotein; MELD: a model for end-stage liver disease; MELD-Na: MELD-sodium; iMELD: integrated MELD; COSSH-ACLF II: Chinese Group on the Study of Severe Hepatitis B-ACLF II score

**Fig. 3:**
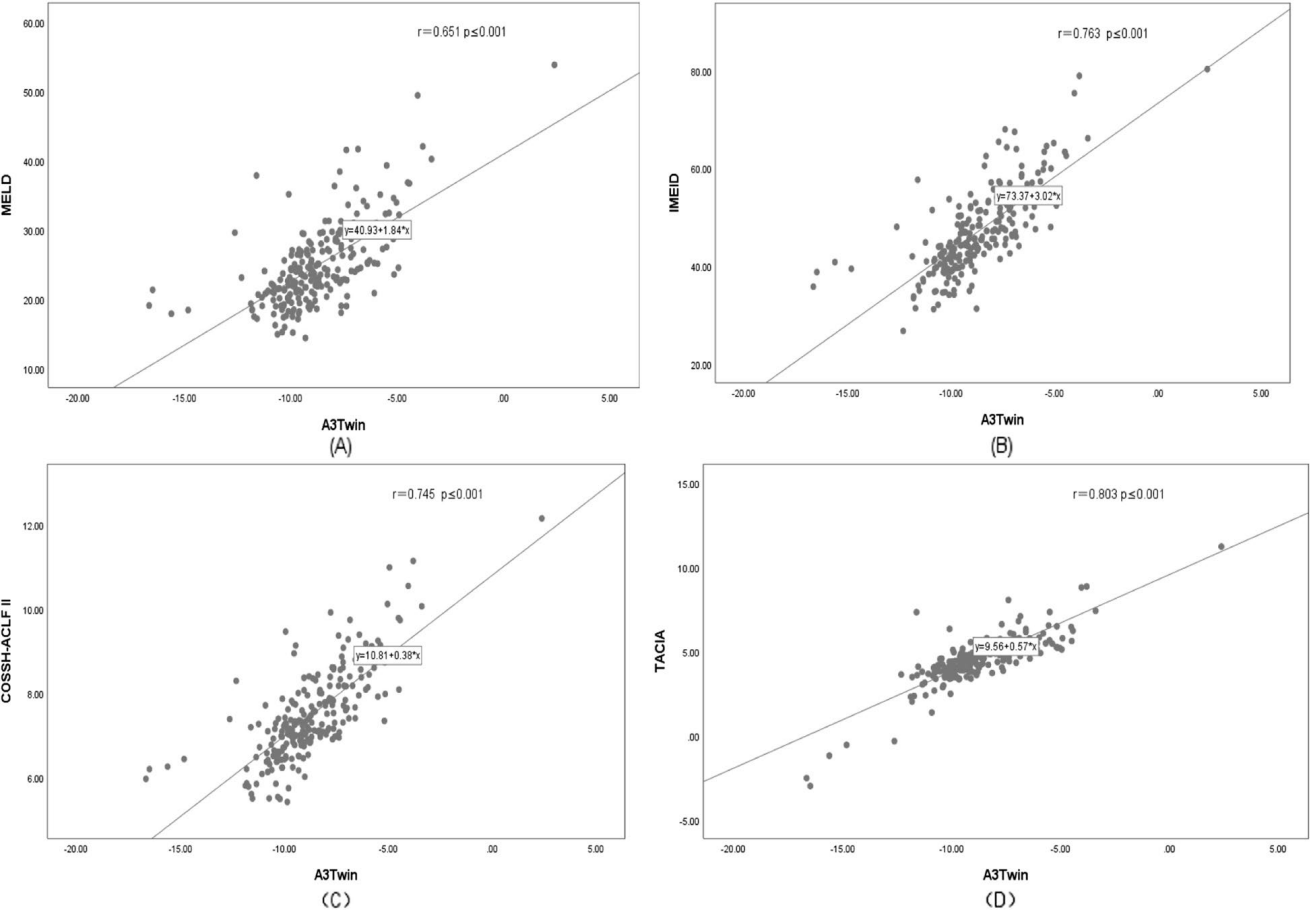
Correlations between the A3Twin score and other prognostic scores. MELDs: a model for end-stage liver disease score; iMELD: integrated MELD score; MELD-Na: MELD-sodium score; COSSH-ACLF II: Chinese Group on the Study of Severe Hepatitis B-ACLF II score

The ROC curves for AFP, the other models, and the new model are shown in Fig. 2. We compared the efficiency of the A3Twin score with the other formulas in predicting short-term prognosis. The ROC analysis showed that A3Twin has good accuracy in predicting mortality [AUC= 0.851, 95% CI (0.801–0.901), *p* < 0.001], followed by the COSSH-ACLF II score [AUC = 0.836, 95% CI (0.784–0.888), *p* < 0.001], TACIA score [AUC = 0.792, 95% CI (0.731–0.837), *p*< 0.001], iMELD score [AUC =0.775, 95% CI (0.713–0.837), *p* < 0.001], MELD score [AUC =0.760, 95% CI (0.697–0.824), *p* < 0.001], and MELD-Na score [AUC =0.723, 95% CI (0.655–0.790), *p* < 0.001]. The results illustrated that the A3Twin score was superior to those models mentioned above. More details are displayed in Table 4. In addition, the A3Twin score was positively associated with the MELD scores (r = 0.651, *P*<0.001) but more strongly associated with the iMEID, COSSH‐ACLF II scores, and TACIA (r = 0.763, 0.745, and 0.803, respectively; all *P* < 0.001; Fig. 3).

**Table 4:** AUC and cut-off values of the prognostic variables and model

**ROC**	**AUC**	**Cut off Value**	**sensitivity**	**Specificity**	**95% CL**	***P* Value**
	**Area**					
A3Twins	0.851	-9.07	0.788	0.781	(0.801,0.901)	<0.001
MELDs	0.760	23.04	0.761	0.676	(0.697,0.824)	<0.001
iMELDs	0.775	46.97	0.646	0.810	(0.713,0.837)	<0.001
MELD-Nas	0.723	25.09	0.726	0.657	(0.655,0.790)	<0.001
COSSH-IIs	0.836	7.40	0.673	0.848	(0.784,0.888)	<0.001
TACIAs	0.792	4.53	0.743	0.771	(0.731,0.837)	<0.001
AFP	0.619	61.68	0.533	0.699	(0.545,0.694)	0.002

**Fig. 4:**
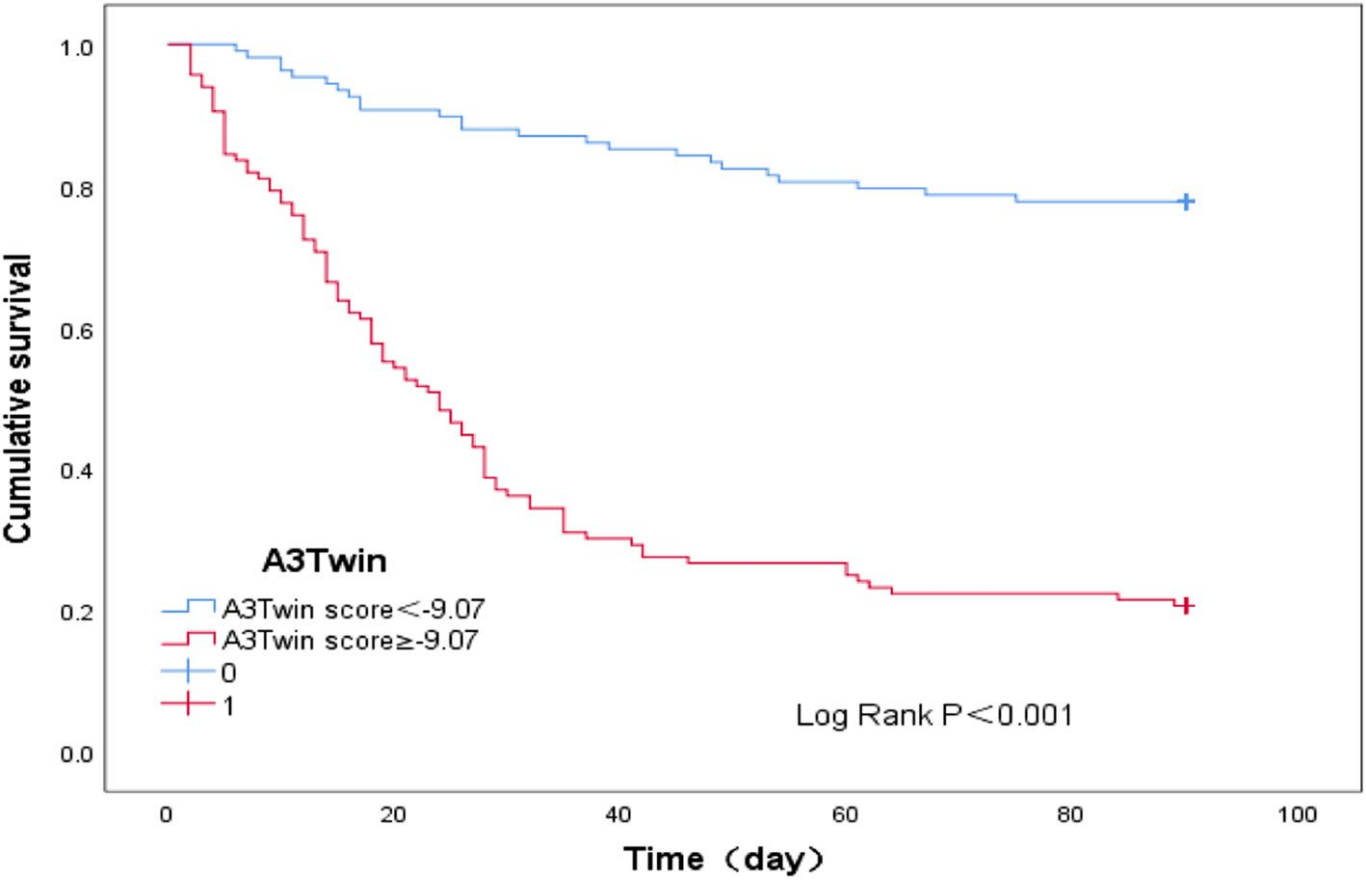
Kaplan‒Meier curve of A3Twin score<-9.07 and A3Twin score ≥-9.07

The applicability of the newly found A3Twin score for predicting a poor prognosis within 90 days in type C HBV-ACLF was shown. A cut-off point of the A3Twin score ≥ -9.07 was suggested to indicate a poor outcome with 78.8% sensitivity and 78.1% specificity. Thus, we further analysed patient survival according to their A3Twin scores (Fig. 4). The transplant-free survival rate at 90 days was 22.40% (28/125) versus 77.80% (79/99) (*P* < 0.001) in the groups of patients with A3Twin scores ≥-9.07 and <-9.07.

## Discussion

ACLF in patients with basic liver disease of different severities has different pathophysiologies, clinical manifestations, and prognoses [20, 21]. Unlike chronic hepatitis, which is more reversible even if it deteriorates into ACLF after an insult, cirrhosis, especially decompensated cirrhosis, is more likely to progress to multiple organ failure, with an “end-stage” irreversible state [22, 23]. In our study, patients with type C HBV-ACLF had a worse prognosis, and the mortality rate reached 52.23%. Consequently, the early identification of patients with type C HBV-ACLF who may not recover from conservative therapies is critical for clinical decisions.

The existing assessment models are based on organ failure and do not account for indicators related to liver regeneration. The secretion of AFP is minimal in an adult liver. Reactivation of AFP production in adults occurs during liver regeneration and hepatic carcinoma genesis [24]. Studies [19, 25] have indicated that elevated AFP levels could predict a better prognosis for HBV-ACLF and acute liver failure with chronic HBV infection. However, a combination of cirrhosis of the liver, acute hepatic insult, and bacterial infection induces severe liver injury with high mortality, recapitulating some features of clinical type C ACLF [8]. Xiang et al. [26] found that chronic liver fibrosis and bacterial infection can suppress liver regeneration due to the shift from the activation of proregenerative IL-6/STAT3 to the antiregenerative IFN-γ/STAT1 pathway in animal experiments. Studies on liver regeneration in type C ACLF are still lacking; therefore, our study explored the prognostic value of liver regeneration in patients with type C HBV-ACLF. We found that the AFP level was significantly negatively correlated with FIB-4 and MLR. From clinical aspects, we demonstrated that acute hepatic injury-induced liver regeneration was markedly suppressed in fibrotic livers, which was also inhibited by inflammation. Whether in clinical studies or animal experiments, PLT has been demonstrated to be able to protect the liver and promote hepatic regeneration [27, 28]. In the present study, we verified that AFP levels were significantly positively correlated with PLTs. We also found that AFP was significantly different between the survival group and the death or transplantation group, which can also be considered a prognostic indicator of type C HBV-ACLF.

Wang et al. [29] showed that liver cirrhosis at admission was an independent risk factor for both short-term and long-term outcomes in HBV-ACLF patients. The severity of liver fibrosis or the degree of portal hypertension plays an important role in the prognosis of type C ACLF. Liver biopsy has been considered the "gold standard" for the diagnosis and grading of liver fibrosis. Measurement of the hepatic venous pressure gradient (HVPG) is currently the best available method and is considered the gold standard for portal hypertension assessment. However, liver biopsy and HVPG measurement are invasive and are only routinely available and/or performed with adequate standards in expert centres. Verma et al. [30] verified that the APRI, which has been proposed as a good noninvasive estimator of hepatic fibrosis, correlates fairly well with the hepatic venous pressure gradient (HVPG) in patients with cirrhosis. Recent studies have detected that noninvasive assessment of liver fibrosis predicted long-term outcomes in patients with chronic HBV and predicted accurately regarding progression to ACLF in patients with AE and SAE [31, 32]. However, the roles of APRI in HBV-ACLF are unknown. In the present study, we found that APRI and FIB-4 were significantly different between the survival group and the death or transplanted group, and logistic regression revealed that APRI was an independent risk factor for prognosis of type C HBV-ACLF.

The other five independent risk factors (TB, INR, age, WBC, Na) were selected, and we developed a simplified A3Twin score for patients with type C HBV-ACLF. Among these factors, TBil and INR were associated with liver and coagulation, respectively, and have been commonly used in previous scores. Livers of the elderly show increased nonparenchymal cell senescence, decreased liver regenerative capacity, altered metabolism functions, and immune response dysfunction, making these patients more susceptible to developing chronic liver diseases, and the prognosis of liver disease is worse [33]. We found that age is closely related to the prognosis of type C HBV-ACLF patients. Additionally, age was significantly associated with the severity of ACLF in both the iMELD, COSH-ACLF, and COSS-ACLF II groups. Systemic inflammation is a major driver of HBV-associated ACLF [34]. Infections are among the most frequent (>30% of patients) precipitating events of ACLF [35], and more than 50% of patients with ACLF develop an infection during their hospital stay, increasing their death rate from 34% to 71% [36]. As an inflammatory factor, WBC was also used in the CLIF-C ACLF score. Diluted hyponatremia is attributed to the impairment of the kidneys to eliminate solute-free water, resulting in a disproportionate accumulation of water with sodium [37]. Ruf et al. [38] found that hyponatraemia is an excellent predictor of outcome in patients with advanced cirrhosis and significantly increases the efficacy of MELD to predict waitlist mortality, establishing MELD-Na. Previous research has expounded that hyponatraemia is a poor prognostic predictor in those with acute-on-chronic liver disease (AoCLD) and ACLF [39, 40]. Hyponatraemia was used in both iMELD and United Kingdom Model for end-stage liver disease (UKELD) scores. Thus, these five independent risk factors reflect type C HBV-ACLF pathophysiology.

Any prognostic score should use objective and accessible clinical indicators to simply and accurately predict the disease outcome for clinical application. The prognostic value for type C HBV-ACLF of our novel scoring system, which was backed by pathogenesis and included seven routine indices, was showed and had an advantage over MELD, iMELD, MELD-Na, COSSH-ACLF II, and TACIA. Patients who have lower A3Tiwn scores (<-9.07) might survive longer than those who have a higher A3Tiwn score (≥-9.07). The results indicate that patients with high A3Twin scores might have serious liver dysfunction, inflammation, and liver cirrhosis, and the capability of hepatic regeneration would be diminished.

Our study has several limitations. First, we retrospectively investigated type C HBV-ACLF patients in a single centre, and the sample size was not large enough to establish a validation cohort. Second, the accuracy of serum markers of liver fibrosis for the degree of liver cirrhosis was potentially affected by severe liver inflammation. The potential benefits of incorporating the APRI into the prognostic model should be further confirmed in another independent and larger study. Third, due to the retrospective study, some indicators could not be collected (such as arterial partial pressure of oxygen and lactic acid). Our new model cannot be compared with the other models (CLIF-C ACLF、 AARC、COSSH-ACLF). Finally, due to regional differences, the etiology of acute-on-chronic liver failure is different in the East and the West, and the definition of chronic liver disease is different. Whether the new model can predict patients with other causes of acute-on-chronic liver failure needs further verification. Type-C HBV-ACLF patients is associated with higher levels of inflammation, fibrosis, and poorer liver regeneration than type A/B HBV-ACLF [8, 21]. Whether the new model which based on liver function, fibrosis, inflammation level, and liver regeneration can predict type A/B HBV-ACLF needs further verification. Large-scale, multicenter, prospective studies are needed to evaluate the usability of this novel prognostic model, especially in patients with other causes or other types of acute-on-chronic liver failure.

## Conclusion

Alpha-fetoprotein and APRI can be used as prognostic indices of patients with type C HBV-ACLF. The novel model, which is comprised of seven routine indices, could effectively predict the prognosis of type C HBV-ACLF. A lower A3Twin score could indicate a better outcome. The results of our research might be helpful in the management of type C HBV-ACLF in clinics.

### Supplementary Information

Supplementary Material 1.

## Data Availability

The data used to support the findings of this study are available from the corresponding author upon request.

## Abbreviations

ACLF: Acute-on-chronic liver failure
WGO: World Gastroenterology Organization
HBV-ACLF: Hepatitis B-related acute-on-chronic liver failure
ROC: Receiver operating characteristic
HCC: Hepatocellular carcinoma
Pre-ALB: Pre-albumin
NLR: Neutrophil to lymphocyte ratio
MLR: Monocyte to lymphocyte ratio
HE: Hepatic encephalopathy
HBV: Hepatitis B viral
HBV DNA: Hepatitis B virus deoxyribonucleic acid
AKI: Acute kidney injury
TBil: Total bilirubin
INR: International normalized ratio
AFP: Alpha-fetoprotein
WBC: White blood cell
Na: serum sodium
APRI: Aspartate aminotransferase/platelet ratio index
AUC: Area under the curve
APASL: Asian Pacific Association for the Study of the Liver
CLD: Chronic liver disease
EASL: European Association for the Study of the Liver
MELD: Model for end-stage liver disease
MELD-Na: MELD-sodium
iMELD: Integrated MELD
COSSH-ACLF II: Chinese Group on the Study of Severe Hepatitis B-ACLF
PLT: Platelet
Cre: Creatinine
ULN: Upper limit of normal
CI: Confidence interval
