# Supplementary material for: Alpha-fetoprotein and APRI as predictive markers for patients with Type C hepatitis B-related acute-on-chronic liver failure: a retrospective study
Source: BMC Gastroenterol. 2024 Jun 4;24:191. doi: 10.1186/s12876-024-03276-x (PMC11151586; doi:10.1186/s12876-024-03276-x)
Supplement: Supplementary file 1 — Supplementary Material 1. [file 12876_2024_3276_MOESM1_ESM.pdf]

# 证　明

兹证明 Springer Nature Author Services 委托中国国际图书贸易集团有限公司(CIBTC)代开中国税务发票。目前双方 的合作方式是:支付宝、银联卡和中国国内银行转账这三种支付方式。

使用支付宝付款的交易,其收款方是:中国国际图书贸易集团有限公司。

使用银联卡付款的交易,其收款方是:瑞购网,该网是中国国际图书贸易集团有限公司旗下网站之一。

使用中国国内银行转账方式的交易,其收款方是:中国国际图书贸易集团有限公司,开户行:中国银行总行营业部,银行账号:778350032951。

不论使用支付宝、银联卡还是银行汇款,税务发票都将由中国国际图书贸易集团有限公司开具。

特此证明!

Springer Nature Author Services
2017 年 10 月

## Certification

Springer Nature Author Services hereby authorizes China International Book Trading Cooperation (CIBTC) to issue Chinese tax invoices (Fapiao) on behalf of Springer Nature Author Services. The extent of cooperation is currently limited to payments by Alipay, UnionPay and China domestic bank transfer.

Payment recipients of Alipay users are CIBTC.

Payment recipients of UnionPay users are Readgo.cn, which is one of the websites that belong to CIBTC.

Payment recipients of bank transfer users are: China International Book Trading Cooperation (CIBTC), bank name: Bank of China, account number: 778350032951.

Regardless of payment method, Alipay, UnionPay or bank transfer, all Fapiao will be issued by CIBTC.

Hereby certified,

Springer Nature Author Services

October 2017
